# The Dengue

**Published:** 1903-08-15

**Authors:** 


### THE DENGUE.

It has constantly been a puzzle to medical men who have had opportunities of studying it in different parts of the world, why in some places the dengue should be so infectious that not a single susceptible individual will escape having an attack, while in other districts it fails to spread at all through the community. Dr. Graham[1] had the opportunity of carrying out an extensive investigation into the cause of the disease and the method by which it was spread during a recent epidemic which occurred at Beyrouth. As a result he has arrived at conclusions which, if subsequently verified, will not only explain the apparent vagaries of the fever, but will also show what must be done in order to prevent it from spreading. Past epidemics in various countries have made prominent the fact that dengue as a rule cannot extend to any great degree amongst communities who live at high altitudes and in districts where water is scarce. Partly for this reason and partly on account of other peculiarities Dr. Graham suspected the mosquito as being the principal agent by which the infective agent was carried. Beyrouth itself is infested with mosquitos. Though careful search was made none of the forms of *anopheles* were found, though *culex fatigans* was present in abundance as was also *stegomyia fasciata*. Attention was therefore directed to *culex fatigans*, with a view to discovering by direct experiment whether it was capable of conveying the contagion.

The first experiment was done with a nursing mother and her child. The day the mother was taken with the initial chill of an attack of dengue all the mosquitos in one room were killed by generating chlorine gas, and each day afterwards she entered another room in which the mosquitos had been killed in the same way, the rooms being protected as well as was possible from the re-entrance of mosquitos. The child was nursed by his mother during the whole time she was ill with the fever, which was a severe and typical attack, and yet escaped any apparent infection. In experiment number two the same plan was adopted with four children, one of whom had contracted the fever. Care was taken to exclude mosquitos from the room in which they were, and although they all slept in the same bed at night none of the other three contracted dengue. The rooms were kept free from mosquitos for thirteen days. The next series of experiments consisted in submitting individuals, who volunteered themselves, to inoculation in the following manner. Four healthy men were selected, inmates of houses where no case of dengue had occurred. Into the mosquito nettings in which these men slept were placed night after night mosquitos which had been collected from within the mosquito nets of persons already suffering from the disease. All the men were confined to their houses during the process, and every precaution was taken to exclude infection from other sources than by the mosquitos. Of these four men three were attacked with typical dengue. The initial chill occurred in their different cases on the fourth, the fifth, and the sixth days respectively, dating from the first night that the mosquitos were admitted. In the fourth case no attack occurred. The man in question had suffered severely with dengue twelve years previously, and Dr. Graham thought he might possibly be immune for that reason. Similar experiments were carried out on two men in a village where no dengue had as yet occurred, and where there were scarcely any mosquitos. Both these men got infected, the one after four nights and the other after five nights with the contaminated mosquitos.

These men continued to sleep under the mosquito-nettings until some time after they were well, all the mosquitos having been killed, and no further cases of dengue occurred in the village. These observations cetrainly appear to go a long way towards indicating the *culex fatigans* as an important carrier of the contagion of dengue. Dr. Graham turned his attention next to the blood of patients suffering from the disease, and here he was able to find in the red corpuscles an organism showing amœboid movement and resembling the parasite of malaria. The method of examining specimens was similar to the procedure generally adopted for examining the blood in malaria. The parasite is found in a much smaller number of corpuscles than is the malaria parasite in an ordinary case of tertian fever, but it may always be found if careful search is made. It is evidently a protozoon, but it differs from the malaria parasite in that it contains no pigment. The parasite was always to be found in the blood in the stomachs of mosquitos which had been removed from inside the mosquito curtains of people ill with the disease, and it was always found up to the fifth day after the mosquito had sucked the blood. Sporulation occurs in the stomach of the mosquitos, and Dr. Graham was able to find spores in the salivary glands of mosquitos within 48 hours of their having sucked the blood of a patient who had been ill with dengue for four days. It is these spores, he believes, by which the human is infected with dengue, the spores being introduced by the mosquito, and subsequently developing in the blood current. One direct inoculation experiment was made. The salivary glands were dissected out from a mosquito with a 27 days' old infection, and were mixed in a warm sterilised normal saline solution. This was then injected subcutaneously. The patient was taken ill on the third day with a sharp attack of dengue, in fact the illness was so severe that the experiment was not repeated. With regard to the other inoculation experiments which he had performed, Dr. Graham remarks that dengue is practically never fatal, and that every susceptible individual is certain to get the disease before the epidemic is over, that is to say, in a place like Beyrouth which is favourable to the spread of dengue.

[1] Journal of Tropical Medicine, July 1.

